# Frailty and deconditioning on the acute take

**DOI:** 10.1016/j.clinme.2025.100548

**Published:** 2026-01-05

**Authors:** Bhagya Arun, Siobhan H.M. Lewis

**Affiliations:** a Internal Medicine Trainee, Health Education and Improvement Wales, Cardiff, Wales, UK; b Department of Clinical Gerontology, University Hospital of Wales, Heath Park, Cardiff, UK

**Keywords:** Deconditioning, Frailty, Emergency care, Acute hospital

## Abstract

It is well known that older patients have a higher incidence of adverse hospital outcomes, such as inpatient falls, delirium and longer lengths of stay, and that older people are likely to become deconditioned during their time in hospital. It is often felt that deconditioning occurs as a consequence of a prolonged stay; however, the adverse effects of immobility can occur within hours of arrival into urgent care. Hospital-aquired deconditioning is in fact a multi-systemic syndrome that manifests in a number of ways outside muscle weakness, including delirium, healthcare-associated infection, new incontinence and falls, all of which can worsen outcomes for patients and lead to prolonged hospital stays. We outline strategies to embed preventing deconditioning into the care of older patients who present to the emergency and acute medical unit in order to avoid unnecessary harm and support patients to maintain their independence.

## Highlights

• Deconditioning occurs rapidly upon arrival into hospital.
• It is associated with delirium, falls, and healthcareassociated infection.
• Promoting early safe mobility is essential.
• Nutrition, hydration, comfort and sleep should be prioritised.
• Deconditioning is everyone’s business, not just frailty teams.

## Graphical abstract

**Figure:**
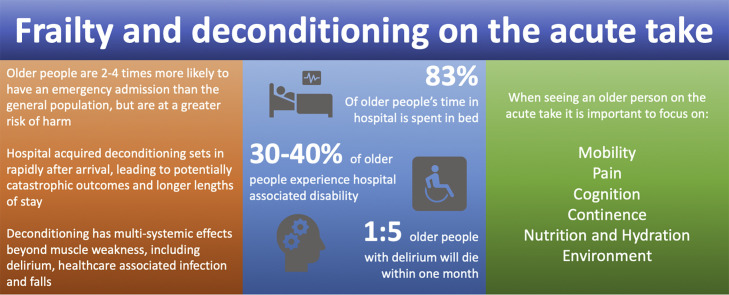

## Introduction

Older people aged 65–84 are twice as likely as the general population to have an emergency admission, increasing to 4–5 times as likely for those over 85, while over 85s have experienced the greatest rise in emergency admissions.^1^ Older people are vulnerable to harm during a hospital stay, in particular to the effects of hospital-acquired deconditioning (HAD).

Deconditioning begins at the onset of acute illness or injury but is greatly exacerbated by processes that occur on arrival into hospital, such a that by the time a person has reached an inpatient ward, deconditioning is well established. Upon arrival to the emergency department (ED), patients are frequently confined to trolleys for extended durations, accelerating physical decline. Factors such as poor sleep, ward moves, difficulty accessing food and fluids, and delirium are common.

It is this initial decline in function that then leads to new disability and functional impairment, with long waits for assessment and discharge planning being a consequence rather than the cause of deterioration. This then impacts patient flow, leading to further crowding in the ED and acute medical unit (AMU).

## What is deconditioning syndrome?

**Fig. 1:**
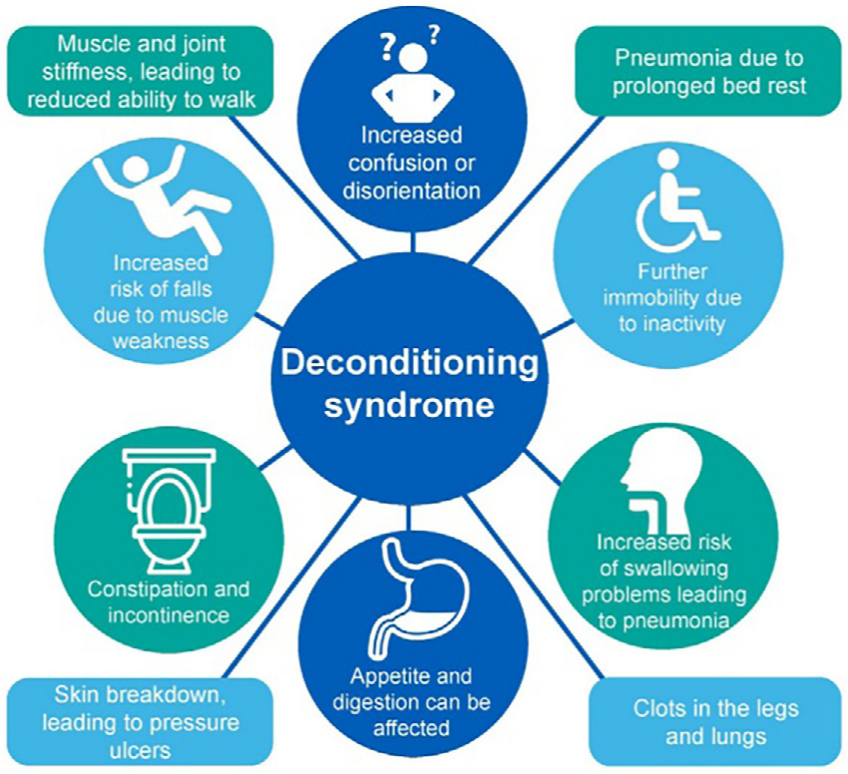
Deconditioning is a multi-system syndrome with wide-ranging symptoms and outcomes. Figure taken from ‘Sit Up, Get Dressed and Keep Moving!’. British Geriatrics Society, University Hospitals of North Midlands NHS Trust (2020). Available at: https://www.bgs.org.uk/%E2%80%98sit-up-get-dressed-and-keep-moving%E2%80%99. Reproduced with permission.^30^

Deconditioning syndrome represents a complex, multi-system decline in physiological function resulting from prolonged immobility, commonly seen in frail patients; however, it can occur in any age group (Fig. 1).

### The impact of deconditioning

**Fig. 2:**
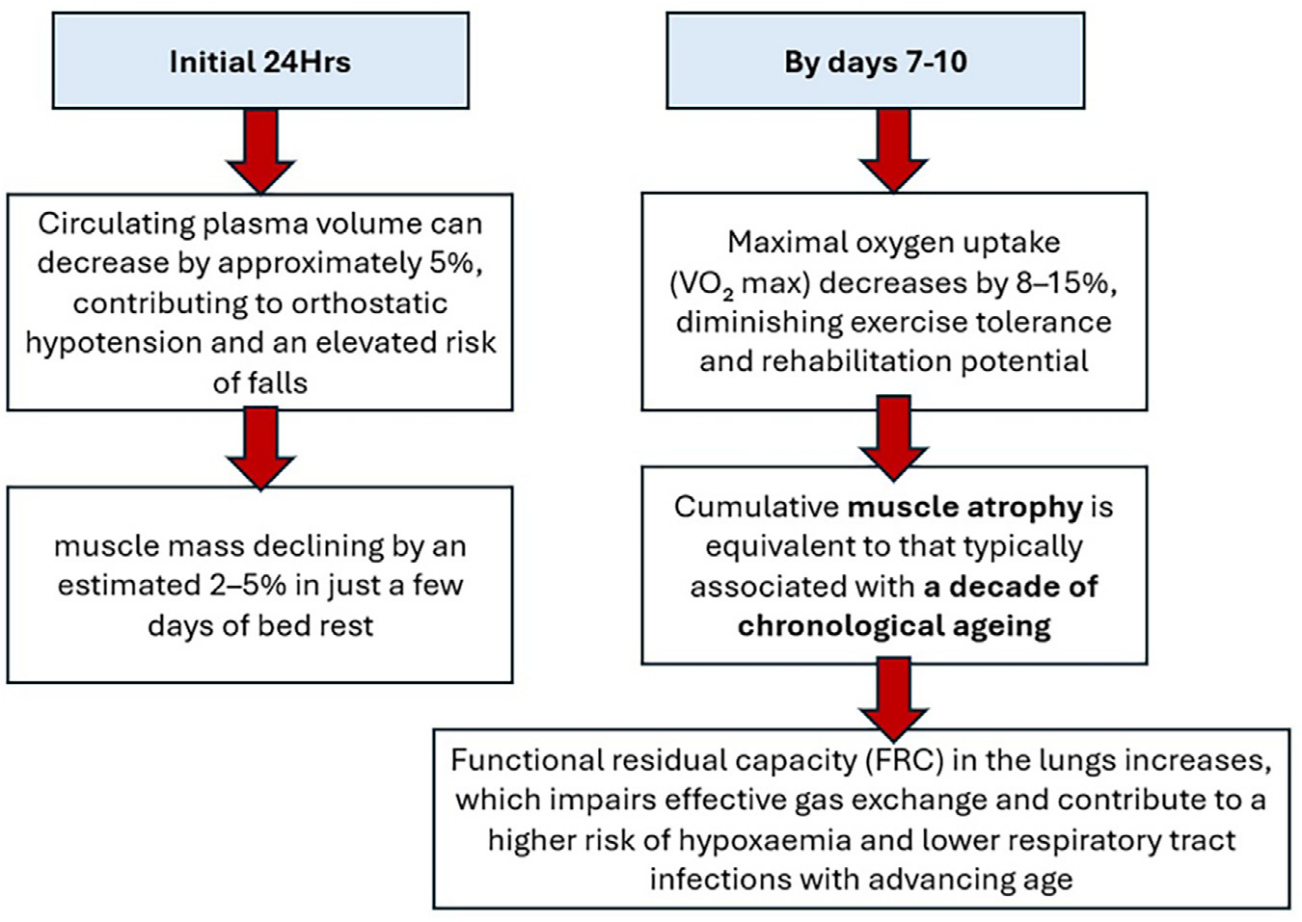
The physiological effects of prolonged immobility (adapted from^31^, ^32^, ^33^, ^34^, ^35^).

Extended periods of bed rest have multi-systemic physiological effects in older people that emerge rapidly during inactivity (Fig. 2) and are associated with significant adverse outcomes (Table 1).

**Table 1:** HAD is associated with adverse hospital outcomes.

The impact of hospital-acquired deconditioning in older people
Hospital-associated disability affects 30–41% of older adults following an acute hospital admission.^36^
Among community-dwelling older people, hospital admission carries a fourfold risk of functional decline.^37^
47% of older adults have their discharge delayed by deconditioning.^38^
Up to 50% of older people develop new incontinence within 48 h of a hospital admission.^39^
In-hospital activity levels are low, with 83% of time spent in bed and 12% in a chair.^40^
Only 30% older people who develop a new disability are back to their usual level of function at 1 year.^41^

## Key focus areas when considering hospital-acquired deconditioning

### Falls

It is understandable that avoidance of inpatient falls is a key patient safety focus. This has, however, inadvertently led to practices that limit activity, leading to muscle weakness, worsening balance, joint discomfort and a greater likelihood of recurrent falls. Encouraging inpatients to be active will increase their exposure to falls, but introducing measures to support people to do so safely will lead to a reduction in the overall risk.^2^

The common misconception that older patients require therapy assessments before mobilising often leads to further immobility and is usually only necessary if there is a specific concern.^3^ Simply asking someone how they usually walk can mean that walking aids can be sourced and patients can quickly regain their usual mobility.

### Pain

Acute fractures are a common ED presentation, and while there is clear guidance for management of hip fracture,^4^ there is much variation in the care of other fragility fractures. Older ED patients are less likely to be offered and will wait longer for analgesia,^5^^,^^6^ worsening immobility and precipitating delirium.^7^ Staff may feel reticent to use opioids; however, adequate analgesia can often hasten cognitive recovery. Using ‘as required’ analgesia can lead to under-dosing, as older people may not request analgesia. In acute fracture, regular analgesia will usually be necessary – usually paracetamol and a low-dose opioid, eg Oramorph 2.5 mg (codeine should be avoided due to it having a less reliable analgesic response when compared with morphine).^8^ Ask about pain *during mobility*, else they may deny pain simply because they have not yet mobilised.

Older adults have a high prevalence of chronic pain,^9^ which is exacerbated by immobility and may lead to new disability in hospital, even if unrelated to the initial presentation. Patients taking long-term opioids may have developed tolerance and require careful prescribing.

### Delirium

Delirium is associated with a threefold increase in mortality,^10^ double the length of stay (LOS),^10^ a twofold risk of inpatient falls^11^ and nearly three times the risk of early mortality.^12^ Up to 40% of delirium cases are, however, preventable.^13^ All older adults should undergo delirium screening using the 4AT in ED/AMU.^14^

Delirium is disorder of attention, presenting as increased arousal or, more commonly, reduced responsiveness (*think: ‘Drowsy = Delirium’*). Presentations such as reduced mobility and poor oral intake are often-missed clinical manifestations of delirium.

**Fig. 3:**
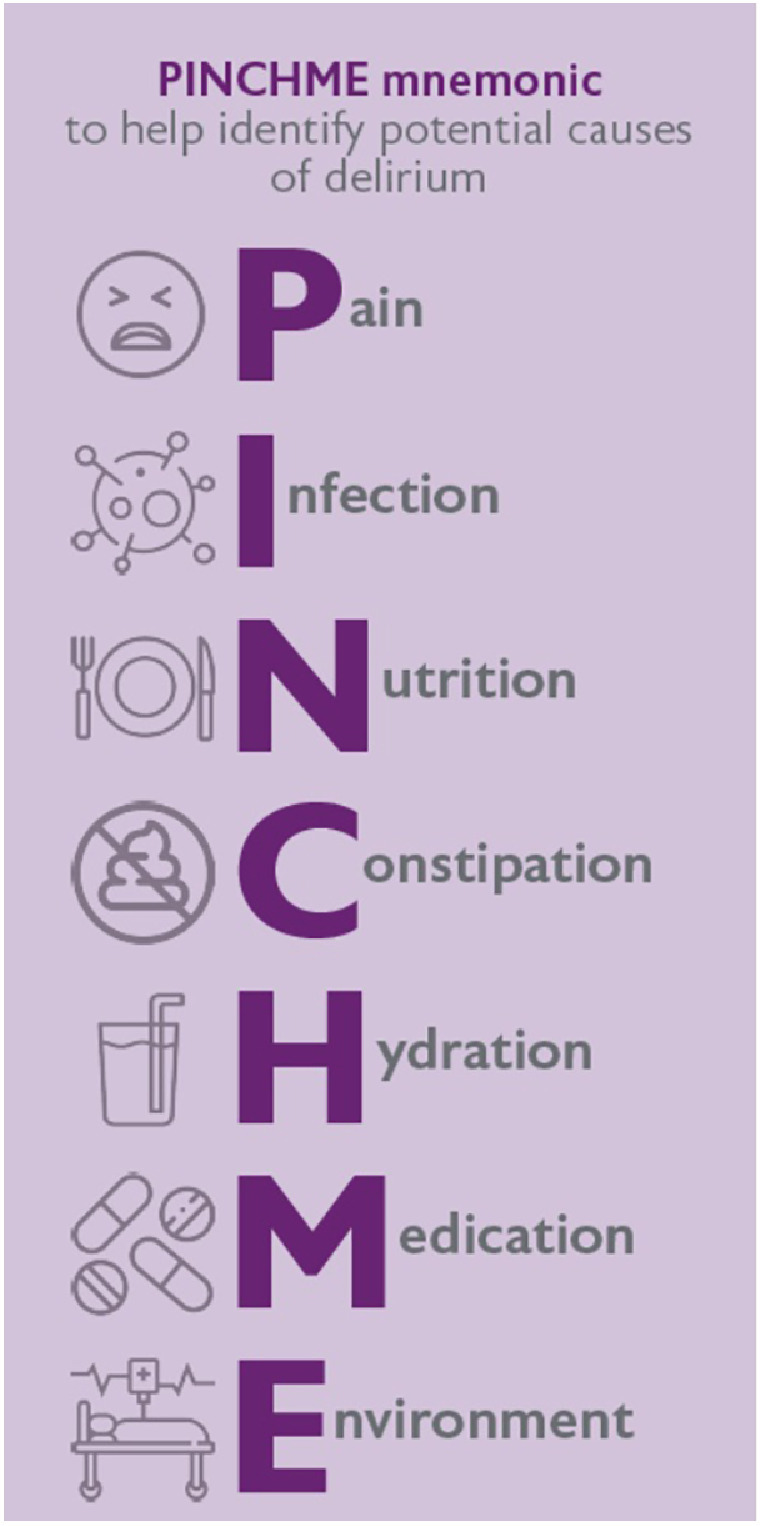
PINCHME mnemonic, Figure taken from End of Life Care in Frailty: Delirium. British Geriatrics Society (2020). Available at: https://www.bgs.org.uk/end-of-life-care-in-frailty-delirium. Reproduced with permission.^42^ Early recognition and addressing these modifiable elements can improve patient outcomes. Any new onset of drowsiness, agitation or confusion in a hospitalised patient warrants a systematic assessment using a framework such as PINCHME to rule out and treat underlying causes of delirium.

Delirium has a range of precipitants, which are often overlooked (see Fig. 3). During the initial hours of an ED/AMU stay, inadequate nutrition and hydration are prevalent and often under-recognised contributors, while constipation is an often-missed cause among inpatients, particularly in prolonged delirium.

Delirium is often attributed to urinary tract infection (UTI); however, this diagnosis should only be made when there are clear symptoms and signs. A ‘strong smell of urine’ is frequently due to either concentrated urine (from dehydration), or the presence of stale urine on clothing. Erroneously attributing delirium to UTI risks overlooking alternative and potentially serious diagnoses.

### Urinary incontinence

New incontinence is predominantly caused by impaired mobility, further exacerbated by patients feeling confined to trolleys or a lack of accessible toilets in ED/AMU. Iatrogenic causes, such as diuretics or intravenous fluids leading to rapid bladder filling, can be further exacerbated by patients feeling immobilised by intravenous lines. Pad use is frequent despite many patients being usually continent. Often once a pad is in place, this is continued throughout admission, leading to reduced independence, further immobility and subsequent new disability. A post-void bladder scan should be performed in all cases of new incontinence to rule out retention presenting as overflow incontinence.

Urinary catheters are associated with infection and delirium, yet up to 65% of urinary catheters inserted in ED may be inappropriate.^15^ Urine output measurement can be undertaken via collecting devices alongside clinical evaluation of hydration status. For essential catheters, leg bags should be used to support mobility, and using valves that prevent free flow of urine can support maintenance of usual continence upon removal.

Bed pans and urinals should be avoided in the ED/AMU. Encouraging patients to walk to the toilet supports both continence and maintenance of muscle strength, balance and functional ability.

### Healthcare-associated infection (HCAI)

Patients who remain in bed experience pooling of secretions which, alongside deconditioning of the respiratory muscles, can lead to hospital-acquired pneumonia. Deconditioning of the swallowing muscles, along with poor oral hygiene, can lead to aspiration. Patients who become pad users during an acute hospital stay have a fourfold risk of developing a hospital-acquired UTI.^16^

### Nutrition and hydration

Hospitalised patients are at increased risk of malnutrition not solely due to illness, but due to reduced activity of the gastrointestinal systems from immobility. This, alongside a change in diet and medications, can lead to constipation and reduced appetite. Access to food and fluid can be challenging in the ED/AMU. Visual, hearing or cognitive impairment may impair awareness that food and fluids are available. Patients with sarcopenia may struggle to position themselves for safe eating and drinking, while lost dentures frequently exacerbate nutritional deficits. Poor oral health quickly leads to malnutrition and dehydration, yet provision of good mouth care is an often overlooked.^17^

Drinks should be offered at every clinical encounter, and food and fluids placed within reach and sightline. Drink and snack trolleys and supporting group mealtimes should be encouraged.

### Environment

Sleep disruption is linked to adverse hospital outcomes including delirium onset, impaired wound healing, increased pain sensitivity and longer hospital stays.^18^, ^19^, ^20^, ^21^, ^22^, ^23^ Patients are frequently woken at night, yet bundling of care to reduce night-time disturbance can enhance sleep quality, reduce sedative and analgesia use, and shorten LOS.^21^ Artificial lighting staying on at night and a lack of natural daylight interfere with the circadian rhythm, exacerbating disorientation, but providing access to natural light during the day can reduce LOS and lower the risk of delirium and falls.^24^

High noise levels, coupled with lost hearing aids and glasses, mean that patients can have difficulty communicating with staff. Interventions such as decibel monitors and felt pads on chair legs can lead to reduction in noise levels in busy ward environments,^25^ while hearing amplification devices should be available in all acute settings.

Clear signage with large fonts, high-contrast colours and pictorial cues can help patients with cognitive or visual impairments. Furniture should be of suitable height to support safe transfers. Patterned floors should be avoided as they can be distracting for patients with cognitive and visual impairment, who may perceive these as objects to walk around.^26^ Spaces that encourage social interaction and therapeutic activity can also contribute positively to emotional wellbeing and cognitive stimulation.^27^^,^^28^

## What next for preventing deconditioning?

Campaigns such as ‘End PJ paralysis’ that promote prevention of deconditioning in hospital settings have demonstrated success in reducing inpatient falls, LOS and discharge to someone’s own home.^29^

The Welsh Six Goals for Urgent and Emergency Care Programme has promoted preventing deconditioning as one of its core principles of optimal hospital flow, emphasising how reducing HAD can lead to better outcomes across the whole system.

Deconditioning prevention should not be siloed as good practice in a few enthusiastic ward areas, but an ethos that ‘*preventing deconditioning is everyone’s business*’, and a culture that promotes independence from time of arrival must be embedded system-wide.

## Conclusion

HAD involves multiple body systems and has significant adverse outcomes including increased falls, HCAI and delirium. Patients must be supported to maintain their independence from the moment of arrival. Campaigns that promote mobility can improve outcomes for patients, but it is important that these are embedded into the core culture of emergency care, and that deconditioning is considered to be ‘*everyone’s business’*.

## CRediT authorship contribution statement

**Bhagya Arun:** Writing – original draft. **Siobhan H.M. Lewis:** Writing – review & editing, Conceptualization.

## Declaration of competing interest

The authors declare the following financial interests/personal relationships which may be considered as potential competing interests: Siobhan H M Lewis reports a relationship with NHS Wales Performance and Improvement that includes: employment. If there are other authors, they declare that they have no known competing financial interests or personal relationships that could have appeared to influence the work reported in this paper.
